# Investigating the effect of a nap following experimental trauma on analogue PTSD symptoms

**DOI:** 10.1038/s41598-021-83838-1

**Published:** 2021-02-25

**Authors:** Ines Wilhelm, Yasmine Azza, Karin Brennwald, Yamina Ehrt-Schäfer, Erich Seifritz, Birgit Kleim

**Affiliations:** 1 Department of Psychiatry, Psychotherapy and Psychosomatics, Psychiatric Hospital, University of Zurich, Zurich, Switzerland; 2 Department of Psychology, University of Zurich, Zurich, Switzerland; 3 Department of Psychiatry and Psychotherapy, University of Lübeck, Lübeck, Germany

**Keywords:** Psychology, Cognitive neuroscience, Emotion, Learning and memory, Stress and resilience

## Abstract

Cognitive models assume that the incomplete integration of a traumatic experience into the autobiographical memory results in typical symptoms associated with post-traumatic stress disorder (PTSD) such as intrusive re-experiencing. Sleep supports the integration of new experiences into existing memory networks through memory consolidation. In fifty-six females, we investigated whether a 90-min daytime nap (n = 33) compared to a wake period (n = 23) after being exposed to an experimental trauma (i.e. a trauma film) prevents PTSD analogue symptoms. Intrusive memories were recorded for seven days using a diary, overall PTSD symptoms were assessed using the Impact of Event Scale (IES-R) and affective response to trauma cues were measured one week after experimental trauma. The two groups did not differ in any of the analogue PTSD symptoms. However, participants obtaining rapid eye movement (REM) sleep in the nap experienced less distressing intrusive memories. Moreover, the duration of REM sleep and slow wave activity was negatively correlated with analogue PTSD symptoms. Our findings suggest that even a short sleep period after experimental trauma can play a protective role in trauma memory formation but only if the nap contains REM sleep. Our data provide additional evidence for a critical role of REM sleep in PTSD development.

## Introduction

In western societies, the lifetime prevalence for experiencing a traumatic event is 30–70%^1^. Only 8–15% of trauma survivors develop post-traumatic stress disorder (PTSD) in response to this traumatic experience^2^. During the last years, a growing body of research aimed to uncover factors that are associated with a higher risk of developing PTSD. In this context, one field of research focused on the role of sleep in the early aftermath of a trauma^3,4^. Sleep disturbances after a traumatic event are frequent, and they are indeed associated with negative long-term outcomes^5,6^. Moreover, those trauma survivors developing PTSD differ from those without PTSD by a significant reduction and fragmentation of rapid-eye movement (REM) sleep^7–10^ as well as increased activity of the aminergic system during REM sleep^7,11,12^. Although a causal role of sleep alterations in PTSD cannot be concluded, these findings provide first hints for a predictive role of sleep in the development of PTSD after experiencing trauma.

According to cognitive models, PTSD has been considered a result of the maladaptive processing of a traumatic event^13–15^. More specifically, characteristic symptoms of PTSD such as intrusive re-experiencing, hyperarousal and avoidance of trauma-related memories and reminders of the trauma are possibly related to the incomplete integration of the traumatic experiences in autobiographical memory networks. The gradual integration of newly acquired experiences in the existing neocortical memories resulting in a stabilized memory trace is a consequence of a process referred to as long-term memory consolidation^16^. A wealth of studies demonstrates that sleep supports the long-term consolidation of new experiences^17,18^. Even a short daytime nap compared to a wake interval following learning resulted in increased performance at a later recall^19,20^. During post-learning periods of sleep, newly acquired memories that are initially stored in the hippocampus become reactivated and thereby integrated into the existing neocortical networks. Slow wave activity (SWA) and sleep spindles which are both characteristic oscillatory activities during non-REM sleep are assumed to play a key role in the consolidation of memories independent of the valence of the memories^17,18,21^. Another line of research suggests a specific role of REM sleep in the processing of emotional memories (see^22,23^ for review). The „sleep to forget sleep to remember” (SFSR) model postulates that REM sleep does, on the one hand, benefit the stabilization of emotional memories and on the other hand, is involved in the reduction of the affective tone associated with the emotional memory^22,24^. Accumulating evidence supports a critical role of REM sleep in emotional memory stabilization (e.g.^25–29^). Few studies also found that REM sleep reduces emotional reactivity associated with a memory^30–32^. However, other studies did not find a REM-related reduction of emotional reactivity but rather point towards a preservation of the affective tone linked to REM sleep^25,33^. Critically, reactivating fear memories during slow wave sleep (SWS) using the method of targeted memory reactivation (TMR) facilitated an attenuated fear response which questions an isolated role of REM sleep in emotional memory processing^34,35^. In sum, these findings from basic research provide insights into possible mechanisms underlying the above-mentioned association between sleep and PTSD. More specifically, healthy sleep might support the adaptive consolidation of traumatic experiences by improving the integration of traumatic experiences into autobiographical memory networks and by reducing the affective tone associated with the traumatic experience.

First empirical evidence suggests that sleep can impact the long-term consolidation of traumatic experiences. In one of our previous studies, we found that a night of sleep compared to a wake interval after experiencing an analogue trauma (i.e. an aversive film) in the laboratory resulted in a lower number and less distressing intrusions in the following week^36^. Another recent study found a negative correlation between REM sleep theta activity in the night after experimental trauma and intrusive symptoms three days later suggesting a predictive impact of REM theta activity in trauma memory formation^37^. In this study, the experimental trauma (i.e. an aversive film) itself was associated with an increase in SWS and a reduction in sleep stage 2 compared to a neutral film condition, independently of later intrusive symptoms indicating a bidirectional relationship between sleep and trauma memories. Moreover, emotional response towards the trauma film was negatively correlated with the duration of REM sleep during the subsequent night^37^.

In the present study, we aimed to investigate (i) the effect of a short daytime nap after an experimental trauma on analogue PTSD symptoms, namely the number of intrusions, associated distress, and overall PTSD symptoms in the subsequent week as well as emotional reactivity to trauma reminders one week later and (ii) the predictive role of specific sleep parameters in a nap following experimental trauma on the development of analogue PTSD symptoms.

## Methods

### Participants

**Figure 1:**
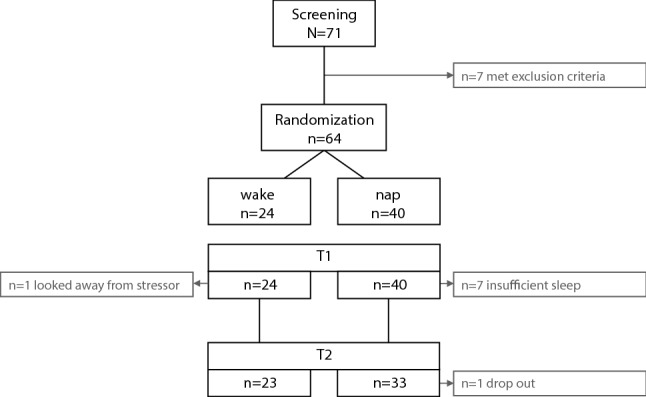
Flow chart of sample size reduction.

Seventy-one female participants aged between 18 and 35 years. were recruited via mailing lists as well as an online platform of the University of Zurich. Only female subjects were included in the study to control for sex differences regarding the emotional response to the film as we expected females to show a much higher response to this type of film (i.e. interpersonal and sexual violence against a women) than males (comparable to previous studies using the same paradigm e.g.^36^). Participants were excluded if they fulfilled one or more of the following criteria: (1) Experience of traumatic events involving interpersonal violence or other traumatic experiences (assessed using adapted version of the Life-Event-Checklist^38^), (2) habitual alcohol or cannabis consumption, (3) frequent watching of violent movies, (4) neurological diseases and/or operations interfering with EEG measurements during the past 6 months, (5) intake of any medication (except vitamins and oral contraceptives) 7 days prior the experiment, (6) diagnosed clinical sleep disorder or self-reported sleep problems and/or self-reported inability to sleep well at new places (Pittsburgh Sleep Quality Index^39^ and Epworth Sleepiness Scale^40^). (7) Diagnosed psychiatric disorder and/or current psychotherapy and/or increased depressive or anxiety as indicated by standardized questionnaires (Beck-Depression-Inventory^41^, Beck Anxiety Inventory^42^ and State Trait Anxiety Inventory^43^). All criteria were evaluated via a telephone interview and a battery of baseline questionnaires before the first experimental appointment. For exact numbers of exclusions please see Fig. 1. Importantly, the nap and the wake group did not differ regarding all measures of psychopathology as well as measures of sleep and chronotype (all *p* > 0.10, see Table 1 for descriptive data).

**Table 1:** Participants’ data.

	Nap (N = 33)	Wake (N = 23)	*t*(*df*)	*p*
	Mean ± SEM	Mean ± SEM		
Age	23.18 ± 0.65	23.96 ± 0.77	− .77(54)	.44
**Questionnaires**				
BDI	3.85 ± 0.77	2.74 ± 0.54	1.08(54)	.28
BAI	4.12 ± 0.78	3.00 ± 0.63	1.04(54)	.30
STAI-T	37.36 ± 1.61	34.04 ± 1.51	1.44(54)	.16
PANAS PA	3.45 ± 0.13	3.30 ± 0.11	0.87(54)	.39
PANAS NA	1.40 ± 0.09	1.30 ± 0.08	0.83(54)	.41
ESS	9.85 ± 0.41	10.09 ± 0.71	0.31(54)	.76
PSQI	3.18 ± 0.24	4.17 ± 0.52	1.73(31.67)	.09

All parameters are given in mean (± SEM) and statistical differences between groups are indicated using independent *t* tests.

*BDI* Becks Depression Inventory, *BAI* Becks Anxiety Inventory, *STAI-T* State-Trait Anxiety Inventory-Trait, *PANAS* Positive and Negative Affect Schedule, *PA* Positive affect, *NA* Negative affect, *ESS* Epworth Sleepiness Scale, *PSQI* Pittsburgh Sleep Quality Index.

Importantly, one participant had to be excluded because she indicated to have looked away “very often” during trauma film presentation. Seven participants of the sleep group had to be excluded due to disturbed sleep during the nap (wake time after sleep onset > 20 min or wake time after sleep onset > 30% of the total sleep time (TST): N = 4; more than 3 awakenings during the nap: N = 3). The remaining 56 participants (sleep group n = 33, wake group n = 23) were included in the analysis of the behavioral data. One additional participant did not attend T2 and had to be excluded from the respective analysis. Two additional subjects had to be excluded for analysis of heart rate at T2 due to artifacts.

Participants received 105 swiss francs (sleep group) or 80 swiss francs (wake group) as compensation for taking part in this study. The study was approved by the local ethics committee (Ethics Committee of the Philosophical Faculty of the University of Zurich) and procedures were carried out in accordance with the approved guidelines. Participants gave written informed consent before participating.

### Procedure

**Figure 2:**
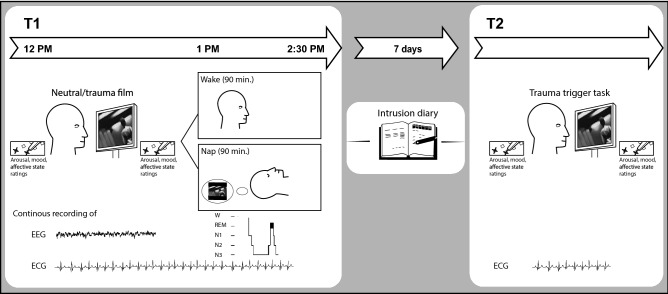
Experimental procedure. In session 1 (T1), participants were exposed to a neutral and a traumatic film. Thereafter, half of the participants were allowed to take a 90-min nap while the other half stayed awake. Before and after each of these films, participants had to indicate their subjective mood, arousal as well as their current affective state. Electroencephalogram (EEG), electromyogram (EMG), electrooculogram (EOG) and electrocardiogram (ECG) were recorded during film viewing (in both groups) and during subsequent sleep (in the nap group only). EEG was recorded from Fz, Cz, Pz, Oz, C3, and C4 (according to the International 10–20 System, referenced to Cz). At the end of T1, participants received the intrusion diary and were asked to fill it in for the following seven days (number of intrusions). During T2, participants were exposed to trauma and neutral film cues, i.e. single pictures taken from the traumatic and the neutral film (i.e. trauma trigger task). ECG as well as subjective measures of mood, arousal and affective state were assessed before and after the trauma trigger task to indicate emotional response toward the film cues. Our main outcomes for the analogue intrusions were (1) number of intrusions (measured by the diary), (2) Distress associated with intrusions (diary) and (3) overall posttraumatic symptom score (IES-R). Further outcomes were the individual change of (4) mood, (5) arousal, (6) heart rate and (7) negative affect in response to trauma cues at T2.

An illustration of the study design can be found in Fig. 2. The procedure consisted of two sessions (T1, T2) separated by one week. Prior to viewing the film, participants were assigned to a sleep or a wake group using a randomization list that was defined beforehand. Participants in the sleep group were adapted to polysomnographic recordings via a daytime nap 2–7 days prior T1. The nap lasted approximately 90 min preceding the actual experiment in order to habituate to sleeping in the laboratory with the scalp electrodes. In the first experimental session (T1), electrodes for polysomnographic recordings (electroencephalogram (EEG), electrooculogram (EOG), electromyogram (EMG) as well as electrocardiogram (ECG)) were placed. Thereafter, participants were seated in front of a monitor in an experimental room, then they were left alone to watch the neutral film followed by the trauma film. The neutral film always preceded the emotional film to prevent spillover effects from the emotional to the neutral film similarly as done in prior studies (e.g.^44^). Participants were instructed to look at the screen continuously. Before and after watching each of the films, participants had to indicate their subjective mood and arousal as assessed by two visual analogue scales (SAM scale^45^) as well as their current affective state as assessed by a short version of the international Positive and Negative Affect Scale^46^ (i-PANAS-SF). The sleep group was then allowed to take a nap for a maximum of 90 min. EEG analysis revealed sufficient sleep quality in the nap group when compared to previous nap studies that were conducted in a sleep laboratory setting (e.g.^47,48^). See Table 2 for descriptive data on sleep parameters. The wake group stayed awake during the same time interval while they were allowed to read, have a snack, or take a short walk. After the retention interval, all participants were given detailed instructions about the intrusion diary they had to fill in for the following seven days. Participants came back to the sleep laboratory for the second session (T2) and electrodes were placed for heart rate recordings (ECG). Participants had to fill in the revised version of the Impact of Event Scale^37^ (IES-R) for a retrospective assessment of subjective symptom severity caused by the trauma film within the preceding seven days. Thereafter, participants were exposed to trauma film cues, consisting of pictures taken from the traumatic film (i.e. trauma trigger task, see a detailed description of the task below). Subjective measures of mood, arousal, and affective state were assessed again before and after the trauma trigger task to indicate emotional response toward the film cues.

**Table 2:** Sleep parameters (*N* = 33).

	Absolute time in minutes (in min)	Percentage of total sleep time (in %)
	Mean ± SEM	Mean ± SEM
Sleep latency	21.21 ± 2.36	
REMS latency	61.42 ± 2.99	
SWS latency	16.68 ± 2.11	
Wake	4.03 ± .07	7.32 ± 1.39
Non REMS 1	4.30 ± 0.61	8.03 ± 1.32
Non REMS 2	28.53 ± 2.77	45.98 ± 3.22
Non REMS 3	19.42 ± 2.81	28.41 ± 3.72
REMS	7.74 ± 1.68	9.74 ± 2.10
TST	64.42 ± 4.19	

*REMS* REM sleep, *SWS* Slow wave sleep, *TST* Total sleep time.

### Trauma film paradigm

The trauma film paradigm was used in a number of previous studies^36,49,50^ and provides a valuable experimental tool for investigating memory processes underlying PTSD with high ecological validity (for review^51^). The trauma film contained a 12 min scene from the movie “Irreversible”, directed by Gaspar Noe depicting a scene of explicit sexual violence. The neutral movie was a film of equal length containing scenes from a National Geographic compilation about blue whales. Participants viewed the films alone in a darkened room without the presence of the examiner and were instructed to pay full attention to the films. After trauma film presentation, all participants were asked on a 5-point rating scale whether and if so, how often they had looked away from the screen (1 = never; 3 = sometimes; 5 = very often).

### Trauma trigger task

High emotional arousal in response to trauma-related cues is a major symptom in PTSD. In order to test the affective response to film-related cues, participants viewed 11 pictures taken from the aversive film each lasting for 2 s. Participants were also exposed to 11 pictures taken from the neutral film after a short break of 5 min. Before and after viewing both picture sets, participants were asked to rate their subjective mood and arousal measured by two visual analogue scales as well as their current affective state measured by a short version of the International Positive and Negative Affect Scale^46^ (i-PANAS-SF). Heart rate was continuously measured during both picture set presentations.

### Intrusion diary

Participants were asked to fill in a pen-and-paper intrusion diary for seven days after being exposed to the trauma film. Whenever they experienced an intrusion during these days, they had to provide a short description of the intrusion and a rating of their subjective level of distress induced by the reported intrusion on a 9-point scale (for a detailed description of the intrusion diary see^36^). The cumulative number of intrusions as well as the associated distress was used as an outcome measure in our analyses.

### Sleep EEG and heart rate

During sleep, EEG, EMG and EOG recordings, and ECG were recorded using a portable amplifier (SOMNOscreen EEG 10-20, Somnomedics, Germany). Participants were not able to see their polysomnographic recordings. EEG was recorded from Fz, Cz, Pz, Oz, C3, and C4 (according to the International 10-20 System, referenced to Cz). EEG signals were sampled at 256 Hz and filtered between 0.016 and 35 Hz. For further EEG analyses which were performed using the BrainVision Analyzer 2.0 (Brain Products, Gilching, Germany), recordings were re-referenced to the mean activity of both mastoids. Power spectral analysis of the EEG signal was performed using Fast Fourier Transformation on all recording sites and separately for periods of non- REM sleep (stages 2 and 3) and REM sleep. The spectra were calculated for successive 8-s artifact-free intervals (2048 data points) using a Hanning window to taper the data. Power density (µV^2^/Hz) was computed for three frequency bands of interest, i.e., for SWA (0.5–4 Hz), theta activity (5–8 Hz), and spindle activity (10–15 Hz). Average power for these bands was calculated first over all bins of the frequency range of interest; then averages were calculated for the succeeding 8–second intervals. Central Electrodes (CzC3C4) were averaged for the main analyses. For sleep stage analysis, recordings were exported from the BrainVision Analyzer and visually scored offline according to standard criteria of the American Academy of Sleep Medicine^52^ (AASM).

Heart rate was analyzed using the “ECG markers” solution in Vision Analyzer 2.0 (Brain Products, Gilching, Germany). All detected R-waves were visually inspected in order to detect and correct artifacts. Mean heart rate in beats per minute was calculated for different intervals of interest (i.e. during exposure of trauma film and neutral film and during associated image presentation as part of the trauma trigger task).

### Statistical analysis

All statistical analyses were performed using SPSS v.23 (IBM Corp., Armonk, NY, USA). Analysis of the effect of sleep on the processing of the trauma film was based on analysis of variance (ANOVA) including the factor ‘group’ (sleep/wake or REM/ noREM/ wake). An additional factor ‘time’ (days 1, 2, 3, 4, 5, 6, 7) was included for analyzing the impact of sleep on intrusion development across seven days. For comparing the response towards trauma film cues at T2 in the experimental groups, an additional factor ‘pre-/post-cue’ (pre-cue: prior to exposure of trauma film cues, post-cue: after exposure of trauma film cues) was included. The affective response towards the trauma film was analyzed using 2 × 2 ANOVA including the factor ‘group’ and either the factor ‘pre- /post-film’ for the dependent variables mood, negative affect and subjective arousal or the factor ‘film type’ for the dependent variable heart rate. Homogeneity of variances was tested using Levene’s Test. In case the assumption of homogeneity was violated Welch ANOVA was calculated which was the case for distress associated with reported intrusion in the three groups: REM, noREM, wake. When appropriate (omnibus *p* value < 0.05), post-hoc tests were performed thereby taking into account multiple comparisons: Tukey HSD post ANOVA and Dunnett T3 after Welch-ANOVA. Effect sizes were calculated for significant results. Correlation analyses were conducted using Pearson's correlation coefficient. In view of the multiple comparisons, a correction of the type I error was applied for statistical analysis concerning correlation coefficients (Šidák correction^53^). The level of significance was set to *p* ≤ 0.05.

## Results

### Affective response to the experimental trauma and trauma memory formation

In a first step, using two-way ANOVAs, we analyzed whether being exposed to the trauma film induced a significant affective response as indicated by various subjective and objective measures. More specifically, we compared negative affect, subjective arousal and mood before and after the trauma film as well as heart rate during trauma film exposure with heart rate during neutral film exposure. We found that negative affect and subjective arousal were significantly increased while negative mood was significantly reduced after trauma film exposure (main effect ‘pre-/post-film’: all *p* < 0.001, see Table 3 for statistical values). Mean heart rate during trauma film exposure was higher as compared to heart rate during neutral film exposure (main effect of ‘film type’: *p* < 0.001, see Table 3 for statistical values). Importantly, there was no significant effect of the factor ‘group’ (sleep/wake) on any of these measures of immediate emotional response to the trauma film (all *p* > 0.12, see Table 3 for statistical values).

**Table 3:** Emotional response to the film material.

	Nap		Wake		Factors		
	Pre film	Post film	Pre film	Post film	‘Pre- /post-film’	‘Group’ (sleep/wake)	‘Pre-/post-film’ × ‘Group’
Negative affect (PANAS)	1.07 ± 0.03	3.09 ± 0.15	1.10 ± 0.04	2.81 ± 0.18	F(1,54) = 251.75, *p* < 0.001,$\eta_{p}^{2} = .82$	F(1,54) = 1.05, *p* = .31	F(1,54) = 1.75, *p* = .19
Mood (SAM scale)	2.36 ± 0.23	6.24 ± 0.32	2.45 ± 0.26	5.55 ± 0.43	F(1,53) = 152.17, *p* < 0.001,$\eta_{p}^{2} = 74$	F(1,53) = 0.75, *p* = .39	F(1,53) = 1.95, *p* = .17
Arousal (SAM scale)	2.42 ± 0.23	6.82 ± 0.32	2.59 ± 0.28	6.18 ± 0.43	F(1,53) = 160.91, *p* < 0.001,$\eta_{p}^{2} = .75$	F(1,53) = 0.53, *p* = .47	F(1,53) = 1.63, *p* = .21

	Neutral film	Emotional film	Neutral film	Emotional film	‘Film type’	‘Group’ (sleep/wake)	‘Film type’ × ‘Group’
Heart rate (BPM)	76.6 6 ± 2.27	72.65 ± 1.77	77.41 ± 1.70	80.87 ± 2.30	F(1,51) = 14.81, *p* < 0.001,$\eta_{p}^{2} = .23$	F(1,51) = 2.56, *p* = .12	F(1,53) = 0.08, *p* = .77

*BPM* beats per minute, *PANAS* Positive and Negative Affect Schedule, *SAM* Self-Assessment Manikin.

### Effect of napping on processing of experimental trauma

In order to disentangle whether a short nap after exposure to a trauma film affects the processing of experimental trauma we compared in the nap and wake group (1) two measures of intrusive re-experiencing in the week after film exposure namely the number of intrusions and the level of distress associated with these intrusions both recorded using the diary, (2) an overall score for analogue PTSD symptoms as indicated by the IES-R and (3) four measures indicating the affective response towards trauma-related cues one week after film exposure, namely changes in mood, negative affect, subjective arousal and heart rate after exposure to trauma-related cues.

*Intrusive Re-experiencing* Neither the number of intrusions nor the level of distress differed between the nap and wake group (number of intrusions across seven days (2 (‘group’) × 7 (‘time’) ANOVA): main effect of ‘group’ F(1,54) = 2.04, *p* = 0.16, interaction ‘group’ × ‘time’ F(3.56,192.05) = 0.69, *p* = 0.58; distress: main effect of ‘group’ F(1,54) = 0.31, *p* = 0.58). As expected, there was a rapid decrease in reported intrusions in both groups (intrusions: day 1: 3.0 ± 0.27; day 2: 2.27 ± 0.21; day 3: 1.45 ± 0.15; day 4: 1.16 ± 0.16; day 5: 1.02 ± 0.13; day 6: 0.88 ± 0.14; day 7: 0.91 ± 0.13; main effect of ‘time’: F(3.56,192.05) = 30.71, *p* < 0.001, $\eta_{p}^{2} = .56$).

*Overall PTSD symptom score* The IES-R, a standardized questionnaire of subjective response to traumatic events which was filled in one week after trauma film exposure, did not differ between both groups (main effect of ‘group’: F(1,53) = 0.56, *p* = 0.46).

*Affective response towards trauma-related cues* One week after exposure to the analogue trauma, subjects were re-exposed to trauma film cues and neutral film cues (trauma trigger task). The affective response towards these cues was assessed. Participants showed an increase in negative affect, negative mood, and subjective arousal after being exposed to trauma film cues (main effect of ‘time’ (pre-/post-cue): negative affect: F(1,53) = 38.43, *p* < 0.001, $\eta_{p}^{2} = .42$; negative mood: F(1,53) = 91.10, *p* < 0.001, $\eta_{p}^{2} = .62$; subjective arousal: F(1,53) = 47.87, *p* < 0.001, $\eta_{p}^{2} = .48$). No significant main effect of ‘group’ was found: negative affect: F(1,53) = 0.45, *p* = 0.51; negative mood: F(1,53) = 0.93, *p* = 0.34; subjective arousal: F(1,53) = 1.56, *p* = 0.22. Heart rate was higher during exposure to trauma film cues as compared to neutral film cues (F(1,50) = 16.05, *p* < 0.001, $\eta_{p}^{2} = .24$), while no significant main effect of ‘group’ was identified: F(1,50) = 0.01, *p* = 0.95. Importantly, except for the increase in negative mood none of these measures did differ between sleep and wake group (interaction ‘time’ × ‘group’ for negative affect: F(1,53) = 0.06, *p* = 0.81; for negative mood: F(1,53) = 5.43, *p* = 0.02,$\eta_{p}^{2} = .09$; for subjective arousal: F(1,53) = 2.36, *p* = 0.13 or ‘trauma/neutral film’ × ‘group’ for HR: F(1,50) = 0.73, *p* = 40.). The increase in negative mood after being exposed to trauma film cues was higher in the wake group as compared to the nap group (Mean ± SEM; pre-cue: sleep 2.70 ± 0.23, wake 2.68 ± 0.30; post-cue: sleep 3.94 ± 0.30, wake 4.73 ± 0.40).

### Relationship between sleep parameters and processing of experimental trauma

**Figure 3:**
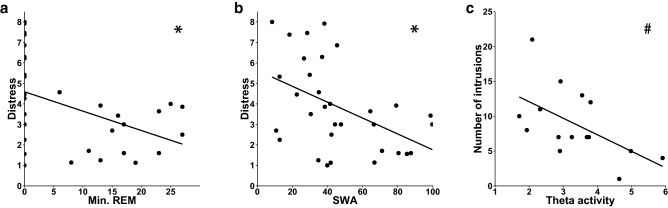
Association between sleep and the processing of an analogue trauma. The amount of REM sleep in minutes (**a**) as well as EEG activity in the slow wave frequency band (**b**) were negatively correlated with the subjective distress induced by reported intrusions. (**c**) There was a tendency for a negative correlation between EEG activity in the theta band (i.e. 5–8 Hz) and the number of intrusions as reported in the intrusion diary cumulative over seven days post experimental trauma. *Significance after Šidák correction; #signifies marginal significance.

In a next step, we analyzed the association between the processing of trauma film memories and sleep oscillatory patterns that had previously been assumed to play a role in the development of PTSD such as REM sleep duration, theta activity in REM sleep, SWA and spindle activity^36,37^. The corrected α level for each family of tests (the three measures of PTSD analogue symptoms namely number of intrusions, intrusion distress and IES-R with each sleep variable) was set to 0.017. REM sleep duration was negatively correlated with subjective distress associated with the intrusions (R =  −0.43, *p* = 0.012; Fig. 3a) but not with the number of intrusions (R =  −0.10, *p* = 0.58), nor the IES-R sum score (R =  −0.15, *p* = 0.43). However, theta activity in REM sleep was negatively correlated with the total number of intrusions across seven days after the trauma film (mean across all central electrodes: R =  −0.56, *p* = 0.03; Fig. 3c) but not with the associated distress (R =  −0.21, *p* = 0.45) or the IES-R sum (R =  −0.38, *p* = 0.16). After correction for multiple testing, the correlation between theta activity during REM sleep and the number of intrusions was only marginally significant. SWA across central sites was negatively correlated with the distress reported in the diary (mean central electrodes: R =  −0.47, *p* = 0.007; Fig. 3b) while no association was found with the number of intrusions (R = 0.03, *p* = 0.86) or the IES-R sum (R =  −0.08, *p* = 0.68) was found.

### Processing of experimental trauma in REM, noREM and wake group

**Figure 4:**
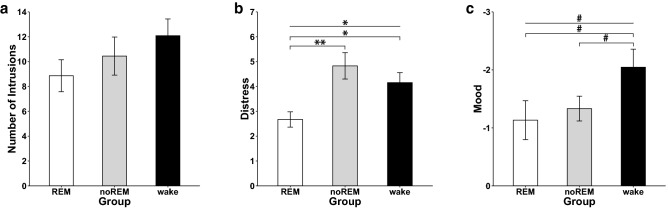
The processing of an analogue trauma in REM, noREM and wake group. The three groups did not differ regarding the number of intrusions as reported in the intrusion diary (**a**). A significant difference (*p < 0.05; **p < 0.01) between REM and noREM was found for subjective distress induced by reported intrusions. The REM group reported a lower level of distress after trauma film exposure as compared to the noREM and the wake group (**b**). The increase in negative mood in response to trauma film cues at T2 was marginally lower (^#^*p* < .1) in the REM and noREM group as compared to the wake group (**c**).

Our previous analysis suggests an impact of REM physiology in the processing of an analogue trauma. In order to consider that only 15 out of 33 participants showed signs of REM sleep during the nap, we calculated additional ANOVAs including the three-way factor ‘group’ (REM, noREM, wake). Again, the number of intrusions in the week after trauma film exposure did not differ between REM, noREM and wake group (main effect of ‘group’: F(2,53) = 1.28, *p* = 0.29; interaction ‘group’ × ‘time’: F(7.09, 187.77) = 0.43, *p* = 0.88; Fig. 4a). However, we found that the three groups differed with regard to the level of subjective distress induced by the reported intrusions (‘group’: F(2,33.98) = 7.96, *p* < 0.001, $\eta_{p}^{2} = .17$; Fig. 4b). More specifically, the subjective distress was lowest in the REM group as compared to the noREM group (*p* = 0.005) as well as the wake group (*p* = 0.017) while there was no difference between noREM and wake group (*p* = 0.49). The IES-R sum score did not differ between the groups (IES-R sum: main effect of group: F(2, 52) = 1.55, *p* = 0.22).

One week after trauma exposure, emotional response towards trauma film cues as indicated by the increase in negative mood differed descriptively, however not significantly between the three groups (interaction ‘group’ × ‘time’: F(2,52) = 2.78, *p* = 0.071, Fig. 4c). Exploratory post-hoc t-tests indicated that the REM group as well as the noREM group showed a marginally lower emotional response to the trauma film cues as compared to the wake group (REM vs. wake group: T(35) =  −1.94: *p* = 0.06; noREM vs. wake group: T(35.65) =  −1.88, *p* = 0.068). Neither negative affect, subjective arousal nor heart rate were differentially affected by the trauma film cues in the three groups (negative affect: F(2,52) = 0.85, *p* = 0.43; subjective arousal: F(2,52) = 1.52, *p* = 0.23; heart rate: F(2,49) = 0.46, *p* = 0.63).

### Relationship between affective response to the experimental trauma and subsequent sleep

As previous studies reported a substantial impact of traumatic experiences on subsequent sleep^9,37^, we examined this association in our sample in an exploratory analyses. Correlation analyses were conducted between the individual affective response to the trauma film and those sleep parameters that have consistently been reported to be affected by traumatic experiences (i.e. sleep latency, total sleep time, amount of REM, SWS sleep, wake time, SWA, REM theta). The increase in negative affect, subjective arousal, heart rate and negative mood in response to the traumatic film was indeed correlated with sleep latency (negative affect: R = 0.42, *p* = 0.015, negative mood: R = 0.46, *p* = 0.007; arousal: R = 0.44, *p* = 0.01). Moreover, REM sleep duration was significantly correlated with the increase in heart rate in response to the trauma film (R =  −0.39, *p* = 0.030). In sum, these findings indicate that the individual affective response to the trauma film predicted sleep latency and REM duration. More specifically, a high affective response to the trauma film was associated with prolonged sleep latency and a lower REM duration during subsequent sleep.

## Discussion

The current study investigated the impact of a nap after exposure to an analogue trauma (i.e. an aversive film) on analogue PTSD symptoms. We further examined the predictive role of several sleep parameters such as REM sleep, REM theta activity, SWA, and spindle activity in the processing of these aversive memories. We found that a nap per se did not affect the processing of an analogue trauma. In line with previous research, we found a negative correlation between REM sleep duration with analogue PTSD symptoms. Because only half of the participants showed signs of REM sleep during the nap, we reanalyzed the data by separating those participants with and those without REM sleep. These analyses revealed a lower level of distress associated with the reported intrusions in the REM group as compared to the wake and the noREM group.

In this study, nap and no-nap groups did not differ in any of the analogue PTSD symptoms which stands in contrast to previous research reporting a protective effect of a night of sleep in the processing of traumatic memories^36^. In fact, a nap is different from a whole night of sleep not only in terms of the absolute amount of sleep stages but also in terms of the distribution of sleep stages. We argue that the small amount of REM sleep during the nap might be one factor explaining the weak effects of sleep in our study. Here, only half of the participants (i.e. 15 out of 33) obtained REM sleep in the nap, and in these fifteen participants, the average proportion of REM sleep of 8% was much lower than what is typical for night sleep (i.e. 20–25% REM sleep). This low amount of REM sleep is an inevitable characteristic of nap study designs resulting from the limited duration of the nap. As REM sleep typically occurs around 70–90 min after sleep onset^54^ a nap significantly restricts the potential time window of experiencing REM sleep. Indeed, the duration of REM sleep was negatively correlated with the distress associated with the reported intrusions as indicated by the intrusion diary. Moreover, there was a marginally significant negative correlation between theta activity during REM sleep and the number of intrusions. Separately analyzing participants obtaining REM sleep during the nap uncovered a beneficial effect of a nap in trauma memory processing. More specifically, the REM group as compared to the wake group showed a lower level of distress induced by intrusions in the week after trauma film exposure and a marginally lower affective response towards trauma film cues at T2. Our findings of a critical role of REM sleep in trauma memory processing is well in line with a recent study reporting a predictive role of REM sleep in the development of intrusive re-experiencing after exposure to a trauma film^37^.

Our findings are also in accordance with the SFSR model (see for review^22,24^). This model postulates that REM sleep does, on the one hand, benefit the stabilization of emotional memories and on the other hand, is involved in the reduction of the affective tone associated with the emotional memory. Apart from the accumulating evidence pointing towards a critical role of REM sleep in emotional memory stabilization (e.g.^25–28,55^), the impact of REM sleep on emotional reactivity is currently controversial (e.g.^25,32,33,55,56^). The fact that we found an association between REM sleep and the reduction in intrusion distress might be related to our specific experimental paradigm. Most basic research studies utilized task material (i.e. emotional pictures showing, for example, a dangerous dog or a gun) that may lack ecological validity as the emotional response towards these kinds of stimuli is only moderate and hence far from being comparable to what is induced by real-life aversive experiences^23^. The aversive film material can induce high emotional arousal which in turn might increase the chance to uncover the depotentiating effects of REM sleep.

The mechanisms behind a possible role of REM sleep in the processing of traumatic memories can be related to the unique neurobiological state of REM sleep. Neuroimaging studies found increased activity in emotion-related brain areas (i.e. amygdala, striatum, hippocampus, insula and medial prefrontal cortex) during REM sleep^57–59^ that might benefit the ability for sleep-dependent reactivation of emotional memories^22^. Importantly, reactivation occurs at the time of low noradrenergic tone in the brain which might result in a decoupling of the affective tone from the emotional memory. Furthermore, high theta oscillations that are characteristic for REM sleep offer the possibility for communication between large-scale brain networks supporting the integration of several distributed aspects of emotional memory. These three biological features of REM sleep are assumed to support the strengthening and integration of the memory while the emotional charge becomes depotentiated over time^22^. However, with the current experiment no final conclusion about REM sleep- related mechanisms is possible as the occurrence of REM sleep was not experimentally manipulated. Future studies are needed that focus on the mechanistic role of REM sleep in trauma memory formation by experimentally manipulating REM sleep. This could be done by either using drugs that are known to alter REM sleep^60^ or by using the natural occurrence of REM sleep, e.g. by comparing REM rich morning naps and afternoon/evening naps.

Apart from the importance of REM sleep, our findings suggest a role of SWS in the processing of an analogue trauma. We found that a high level of SWA is associated with a lower level of distress induced by the reported intrusions. According to the active system consolidation hypothesis, SWA has been assumed to play a key role in reactivating and stabilizing newly acquired memories independent form its emotional valence^17,18,21^. Previous theoretical models pointed towards a sequential role of non-REM and REM sleep requiring multiple of these cycles for a beneficial effect of sleep on emotional memory processing (e.g.^23,24,61,62^). While SWS which typically dominates early periods of sleep may preferentially reactivate and stabilize a memory trace, REM sleep could be of ultimate importance for reducing the affective tone and to finally integrate the traumatic experiences into the existing network of memories. Future studies should scrutinize the sequential and possibly also complementary role of REM sleep and SWA in trauma memory formation.

It is of note, that a significant correlation between emotional reactivity during film viewing and sleep parameters does not provide enough information to infer the direction of effects or even causality. A third variable such as the individual vulnerability could be related to both, sleep parameters as well as the emotional response to the film might increase the PTSD-related symptoms such as intrusive re-experiencing. However, given that previous studies using an experimental design^9,35^ reported similar associations does support our interpretation. Nevertheless, future studies using paradigms experimentally manipulating emotional arousal are needed.

Our study can hardly be compared with previous studies reporting that sleep deprivation after experimental trauma results in reduced analogue PTSD symptoms in the first day after trauma exposure^63,64^. Sleep deprivation does not only affect the consolidation of newly acquired memories but importantly, it immediately disturbs other cognitive processes such as attention, working memory and the ability to retrieve memories^65,66^. Thus, a lower number of intrusions in the first day after sleep deprivation could be a result of these acutely disturbed processes. This notion is also supported by the fact that the number of reported intrusions is lower under acute sleep deprivation but shows an opposite pattern in the days after when participants are no longer sleep-deprived in these studies.

Several limitations have to be mentioned with regard to this experiment. As compared to the wake group, the nap group had the chance for a longer habituation phase due to an additional appointment for a habituation nap. We did not find significant differences in subjective arousal, negative affect, mood and heart rate before and after film exposure indicating that both groups were comparable with regard to baseline stress level and emotional reactivity. Thus, we estimate a potential influence of the adaptation nap to be low even though we cannot entirely exclude it. Importantly, both groups also spent more than one hour in the laboratory before the actual paradigm started (preparation and application of EEG). Upcoming studies should definitely make sure that both experimental groups are habituated to the study environment in the exact same way. Another limitation of the current study concerns the limited control of watching behavior of the participants during the film presentation. All participants included in the analyses indicated not to have consciously looked away from the screen when asked after the procedure. However, subjective ratings are susceptible to response biases. Future studies should make use of objective techniques such as eye tracking to make sure all participants equally draw their attention to the film. Compared to men, women show a greater vulnerability to develop posttraumatic stress symptoms after trauma experiences^67^. Apart from other factors such as the type of trauma and social context the greater prevalence in women has been linked to the impact of sex hormones such as estradiol and progesterone^68^. In our study, we cannot make any conclusions about the role of sex steroids. Future studies need to investigate the influence of sex steroid hormones in trauma processing after an analogue trauma by (1) systematically manipulating the menstrual cycle phase of trauma exposure and by (2) including also male participants.

Our findings provide further support for a possible role of sleep in the processing of aversive memories. Importantly, the majority of mental disorders are not only characterized by maladaptive emotional memories but also by alterations in REM sleep, SWS and/or in spindle activity (e.g.^69–73^). A mechanistic impact of these sleep-related alterations in the development and maintenance of the respective disorders is likely, but far from being entirely elucidated. Here, we demonstrate that studying the role of sleep in the processing of aversive experiences in healthy subjects using the trauma film paradigm can provide novel information on the interplay between traumatic experiences, sleep disturbances, and the development of PTSD. Importantly, future research studying the role of sleep in clinical samples is needed to uncover the clinical relevance of the reported effects. These studies could be a further step towards the development and integration of sleep-related techniques in the treatment of trauma-related stress symptoms.
